# Altered skeletal muscle (mitochondrial) properties in patients with mitochondrial DNA single deletion myopathy

**DOI:** 10.1186/s13023-016-0488-x

**Published:** 2016-07-29

**Authors:** Saskia Maria Gehrig, Violeta Mihaylova, Sebastian Frese, Sandro Manuel Mueller, Maria Ligon-Auer, Christina M. Spengler, Jens A. Petersen, Carsten Lundby, Hans H. Jung

**Affiliations:** 1 Department of Neurology, University Hospital Zurich, Frauenklinikstrasse 26, 8091 Zurich, Switzerland; 2 Institute of Physiology, University of Zurich, Winterthurerstrasse 190, 8057 Zurich, Switzerland; 3 Zurich Center for Integrative Human Physiology (ZIHP), Winterthurerstrasse 190, 8057 Zurich, Switzerland; 4 Exercise Physiology Lab, Institute of Human Movement Sciences, ETH Zurich, Winterthurerstrasse 190, 8057 Zurich, Switzerland

**Keywords:** Bioenergetics, Fat oxidation, Mitochondria, Mitochondrial cytopathy, Neuromuscular disease, Skeletal muscle phenotype

## Abstract

**Background:**

Mitochondrial myopathy severely affects skeletal muscle structure and function resulting in defective oxidative phosphorylation. However, the major pathomechanisms and therewith effective treatment approaches remain elusive. Therefore, the aim of the present study was to investigate disease-related impairments in skeletal muscle properties in patients with mitochondrial myopathy. Accordingly, skeletal muscle biopsies were obtained from six patients with moleculargenetically diagnosed mitochondrial myopathy (one male and five females, 53 ± 9 years) and eight age- and gender-matched healthy controls (two males and six females, 58 ± 14 years) to determine mitochondrial respiratory capacity of complex I-V, mitochondrial volume density and fiber type distribution.

**Results:**

Mitochondrial volume density (4.0 ± 0.5 vs. 5.1 ± 0.8 %) as well as respiratory capacity of complex I-V were lower (*P* < 0.05) in mitochondrial myopathy and associated with a higher (*P* < 0.001) proportion of type II fibers (65.2 ± 3.6 vs. 44.3 ± 5.9 %). Additionally, mitochondrial volume density and maximal oxidative phosphorylation capacity correlated positively (*P* < 0.05) to peak oxygen uptake.

**Conclusion:**

Mitochondrial myopathy leads to impaired mitochondrial quantity and quality and a shift towards a more glycolytic skeletal muscle phenotype.

## Background

Despite progress in understanding the biochemistry and genetics of mitochondrial myopathy, many of the pathophysiological mechanisms remain unclear [1]. Validated therapeutic options as well as simple and effective diagnostic tools are lacking [1–5]. So far, it has barely been investigated how muscle metabolism and morphology may be affected by mitochondrial dysfunction. Fiber type abnormalities including varying distribution of type I and II fibers as well as general or selective atrophy have been reported in patients with various mitochondrial respiratory chain dysfunctions [6–8] but not in adults with mitochondrial myopathy. In a rat model of mitochondrial myopathy [9] a transformation from type I to type II fibers was observed in response to a primary defect of the mitochondrial respiratory chain.

In addition, patients with mitochondrial myopathy have been characterised by lower peak oxygen uptake (*V̇*O_2peak_) and work capacity (*P*_peak_) when performing cycling exercise [10–12]. This limited oxidative capacity in mitochondrial myopathy patients is most likely related to a limited O_2_ extraction with reduced arterio-venous O_2_ difference in exercising muscles [10–13]. The limited O_2_ extraction, in turn, might be explained by an impaired oxidative capacity of the mitochondria itself and/or a reduction in their number [14]. Exercise intolerance can be a prominent clinical manifestation of mitochondrial myopathy, leading to relatively low levels of exertion, fast fatigablity and a sedentary lifestyle [15, 16]. A sedentary lifestyle may further restrict the capacitiy of skeletal muscle for oxidative phosphorylation by a decrease in levels of functional mitochondria [17]. Hence, it remains difficult to distinguish to what extent exercise intolerance is related to impaired mitochondrial oxidative capacity or associated with physical deconditioning. Assessments of muscle oxidative capacity and potential alterations with mitochondrial myopathy have previously only been conducted in single cases or small case series [11]. Thereby, defects of the mitochondrial respiratory chain have been identified polarographically or by enzymatic assays [18, 19], usually involving complex I or III in adults and complex IV in children [8, 20]. Poloarographic measurements have been mainly conducted in complex combinations and not independently for each complex [21]. Moreover, these global assessments of mitochondrial function were conducted in muscle homogenates or isolated fibers [19]. Mitochondrial isolation and homogenation procedures, however, may disrupt the functional network and could thereby alter innate mitochondrial characteristics [22]. Therefore, in vitro measurements of O_2_ consumption in permeabilised myofibers preserving innate mitochondrial function present a more promising approach to determine mitochondrial characteristics [22]. Determination of the respiratory capacity of each individual complex could lead to a better understanding of mitochondrial function and identification of potential mitochondrial-myopathy-related pathomechanisms. So far, the absence of a consistent clinical phenotype has limited the viability of clinical identification and classification criteria of mitochondrial myopathy [3, 21, 23]. However, detailed elucidation of potential pathomechanisms would be important not only for the diagnosis of mitochondrial myopathy but also for developing rational treatment options for mitochondrial myopathy.

Consequently, the primary aim of the present study was to determine mitochondrial volume density and respiratory capacity of each individual complex in permeabilised muscle fibers of mitochondrial myopathy patients by implementing high-resolution respirometry. In our set of patients with moleculargenetically verified mitochondrial myopathy and concomitant exercise intolerance, we hypothesised that mitochondrial volume density and respiratory capacity of each single complex I-V would be decreased compared to age- and gender-matched healthy controls. Furthermore, we aimed to test whether high-resolution respirometry measurements would represent a diagnostic tool leading to enhanced diagnostic and classification criteria of mitochondrial myopathy.

## Methods

### Aim, design and setting

The main aim of the study was to determine mitochondrial volume density and respiratory capacity of each individual complex in permeabilised muscle fibers of mitochondrial myopathy patients and age- and gender-matched healthy controls by the application of high-resolution respirometry measurements. The second aim was to determine if human mitochondrial myopathy with shift towards type II fibers. Additionally, we evaluated if the applied mitochondrial respirometric measurements would represent a diagnostic tool leading to enhanced diagnostic and classification criteria of mitochondrial myopathy.

### Participants

Six patients with mitochondrial myopathy, and eight age-and gender-matched healthy controls, participated in this study (Table 1). Patients were selected from a patient base followed at the Center of Neuromuscular Disorders Zurich (Department of Neurology, University Hospital Zurich, Switzerland) and healthy controls were matched by gender, age and by physical activity patterns (assessed by interrogation). In all patients, clinical and biopsy findings and/or molecular genetic analysis of muscle mitochondrial DNA were in accordance with the diagnosis of mitochondrial myopathy. Apart from histological signs of myopathy and/or serum phosphocreatine kinase (CK) elevation, patients featured a history of muscle weakness, exercise intolerance or exercise-dependent myalgia (Table 2). Prior to the experiments, study participants were clinically examined, exclusion criteria were pregnancy as well as cardiac and/or respiratory disease interfering with exercise tolerance. Several metabolic parameters including plasma lactate, glucose, insulin, leptin, glycated hemoglobin (HbA_1c_), thyroid stimulating hormone (TSH) and CK were measured prio to any study examination and exercise. Cardiac and respiratory health were assessed by 24 h - electrocardiogram, cardiac ultrasound, spirometry and laboratory examination (lipid profile, glucose, precursorprotein brain natriuretic peptide (proBNP)). Patients and controls were neither completely sedentary nor highly active and none of them exhibited diabetes, coronary heart disease, peripheral vascular disease or clinically significant hyperlipidemia.

**Table 1:** Physiological characteristics of patients with mitochondrial myopathy (*n* = 6) and healthy controls (*n* = 8)

	Patients	Controls	*P*-value
Age (yr)	53 ± 9	58 ± 14	0.437
Body mass (kg)	75 ± 18	67 ± 12	0.376
Height (cm)	162 ± 7	167 ± 7	0.238
BMC (kg)	2.3 ± 0.18	2.4 ± 0.4	0.867
Fat mass (kg)	23.1 ± 16.3	14.2 ± 11.6	0.255
Lean mass (kg)	38.4 ± 13.3	41.4 ± 13.9	0.683
Lean mass legs (kg)	19.3 ± 11.1	19.0 ± 8.8	0.951
Total body fat (%)	39 ± 13	31 ± 6	0.153
Relative *V̇*O_2peak_ (mL min^−1^ kg^−1^)	19.8 ± 6.8^a^	32.6 ± 7.3	<0.01
*P*_peak_ (W)	78 ± 21^a^	165 ± 60	< 0.05
[L^−^]_rest_ (mmol L^−1^)	1.64 ± 0.88	1.11 ± 0.62	0.207
RER_25W_ (-)	0.72 ± 0.06^a^	0.69 ± 0.06	0.447
RER_rel_ (-)	0.78 ± 0.08^a^	0.77 ± 0.05	0.923
HR_peak_(min^−1^)	144 ± 28^a^	158 ± 16	0.257
t_lim_ (s)	278 ± 154^a^	541 ± 321	0.118
MVC (Nm)	54.3 ± 41.9^a^	80.6 ± 43.3^b^	0.278

Values are represented as means ± SD. *BMC* bone mineral content; [L^−^], blood lactate concentration at rest; *HR*_*peak*_ peak heart rate, *MVC* maximum voluntary contraction, *P*_*peak*_ peak power; relative *V̇*O_2peak_, peak oxygen uptake per kg body weight; *RER*_*25W*_ respiratory exchange ratio at 25 W, *RER*_*rel*_ respiratory exchange ratio at 50 % *P*_peak_, *t*_*lim*_ time to exhaustion. ^a^ *n* = 5; ^b^ *n* = 7

**Table 2:** Clinical and molecular characteristics of patients with mitochondrial myopathy

Patient	Sex	Age of onset (yr)	Diagnosis	Clinical features	[CK]_rest_ (U L^−1^)	[L^−^]_rest_(mmol L^−1^)	Genetic defect	Mutant mtDNA	Muscle biopsy
1	f	20	CPEO	ptosis, external ophthalmoplegia, facial weakness, slight general weakness	219	normal	mtDNA deletion 4977 bp	> 95 %	COX^−^, ragged blue fibers
2	m	33	CPEO	ptosis, external ophthalmoplegia, general weakness, exercise intolerance	401	normal	mtDNA deletion 4405 bp	90 %	unremarkable
3	f	62	MM	mild proximal lower extremity weakness	normal	normal	mtDNA deletion 5–13 kbp	49–71 %	COX^−^, ragged blue fibers
4	f	12	MM	exercise induced myalgia	206	normal	mtDNA deletion 10–13 kbp	35 %	COX^−^, SDH^+^, ragged red fibers
5	f	20	MM	exercise induced myalgia	normal	normal	mtDNA deletion 6–10 kbp	62 %	ragged red fibers
6	f	39	MM	exercise induced myalgia	normal	5.3	mtDNA deletion 8–12 kbp	12–36 %	unremarkable

[CK], blood creatine kinase concentration at rest; *COX* ^*−*^ cytochrome oxidase negative fibers, *CPEO* chronic progressive external ophthalmoplegia, [L^−^], blood lactate concentration at rest; *MM* mitochondrial myopathy, *mtDNA* mitochondrial DNA, *SDH* ^*+*^ succinate dehydrogenase positive fibers

### Experimental design

Clinical and physiological assessments were performed. Clinical assessments consisted of a neurological examination, muscle strength examination, specific health questions (SF-36 Health Survey), fatigue severity subscale of the Checklist Individual Strength (CIS-fatigue), sensation of pain (visual analogue scale, VAS) and a skeletal muscle biopsy obtained from the *vastus lateralis* muscle. Physiological assessments included a scan for body composition, an incremental cycling test, a constant-load cycling test and measurement of knee extension torque.

### Body composition measurement

Total body mass, bone mineral content (BMC), fat mass, percentage body fat, as well as total lean body mass and lean soft tissue mass of the leg were determined by performing dual-energy X-ray absorptiometry (DXA) measurements using a densitometer (Lunar iDXA^TM^, GE Healthcare, Madison, WI, USA).

### Exercise testing

Each study participant conducted an incremental exercise test on an electrically braked cycle ergometer until volitional exhaustion (Ergoselect 200 K, Ergoline, Bitz, Germany) to determine *V̇*O_2peak_ and *P*_peak_. Additionally, all participants performed a constant-load cycling test at 85 % *P*_peak_ to establish time to volitional exhaustion (t_lim_). Pulmonary gas exchange and ventilation were recorded during both exercise tests using an online gas collection system (Innocor™ M400, Innovision, Odense, Denmark), where flow, O_2_ and CO_2_ concentrations were recorded breath-by-breath. Flowmeter and gas analysers were calibrated prior to and after each test according to the manufacturer’s instructions. Throughout all cycling tests, heart rate was recorded (Polar S610i, Polar Electro, Kempele, Finland) and perceived exertion was assessed via a Borg CR 10 Scale. In addition, blood pressure was registered on a regular basis during the incremental cycling test. The incremental cycling test consisted of a 3 min rest phase followed by 2 min at 25 W with power increments of 25 W every 2 min until volitional exhaustion. Pedal cadences were freely chosen by the participants within a range of 60 and 80 revolutions per minute (rpm) and had to be remained constant throughout the test. *V̇*O_2peak_ was determined as the highest mean over a 10 s period. The respiratory exchange ratios (RER) for submaximal power values were calculated as the mean of the last 30 s at 25 W (RER_25W_) and as the mean over 10 s at 50 % *P*_peak_ (RER_rel_). The constant-load cycling test included a 3 min rest phase, a 3 min warm-up with 1 min at 40 % and 2 min at 60 % *P*_peak_, and a constant-load phase at 85 % *P*_peak_ until volitional exhaustion.

### Isokinetic dynamometry

Knee extensor maximal voluntary contraction torque (MVC) was assessed using an isokinetic dynamometer (Con-Trex MJ, Physiomed Elektromedizin, Schnaittach/Laipersdorf, Germany). Participants’ bodies were stabilised with straps and handles according to the guidelines of the manufacturer. Each participant performed three maximal knee extensions (*ω* = 3.14 rad s^−1^) separated by 1 min rest to assess MVC, whereby only the highest value out of the three trials was used for statistical analysis.

### Skeletal muscle sampling

Participants were tested for coagulation abnormalities (Quick/INR, number of thrombocytes). Skeletal muscle biopsies were obtained under standardised conditions from the *vastus lateralis* muscle under local anesthesia (1 % lidocaine) of the skin and superficial muscle fascia, using the Bergström technique with a needle modified for suction. The biopsy was immediately dissected macroscopically free of fat and connective tissue and divided into sections for actual measurement of mitochondrial respiratory capacity, later transmission electron microscopy (TEM) and histochemistry. The part of the biopsy for the determination of mitochondrial respiratory capacity was directly placed in ice-cold biopsy preservation solution. For TEM, pieces of around 1 mm^3^ of each muscle biopsy were chemically fixed in 2.5 % glutaraldehyde in 0.1 M cacodylate buffer (pH 7.3), stored at room temperature (RT) for 24 h and thereafter at 4 °C until all samples were collected. Muscle tissue for immunohistochemistry was instantly mounted in an embedding medium (Tissue-Tek®, Sakura, Zoeterwoude, The Netherlands), snap frozen in isopentane cooled to −160 °C with liquid nitrogen, and subsequently stored at −80 °C until further processing.

### Mitochondrial respiration measurement

Samples were prepared as described in detail previously [24]. Summarised, after mechanical fiber separation, chemical permeabilization in biopsy preservation solution and washing in mitochondrial respiration medium 05, muscle bundles were blotted dry and measured for wet weight (ww) in a balance-controlled scale (XS205 DualRange Analytical Balance, Mettler-Toledo AG, Greifensee, Switzerland). Respiration measurements were subsequently performed in mitochondrial respiration medium 06. O_2_ consumption of the individual muscle tissue was thereby measured at 37 °C using the high-resolution Oxygraph-2 k (Oroboros, Innsbruck, Austria). Standardised instrumental and chemical calibrations were performed as recommended by the manufacturer and described previously [24]. O_2_ flux was automatically calculated by the software, accounting for nonlinear changes in the negative time derivative of the O_2_concentration signal (DatLab, Oroboros, Innsbruck, Austria). Experiments were performed as duplicates in a hyperoxygenated environment in order to prevent any potential O_2_ diffusion limitation. Thereby, O_2_ concentration ranged between 200 and 450 nmol mL^−1^ within the chambers.

### Respiratory titration protocol

The respiratory measurement protocol was specific to the analysis of individual aspects of respiratory capacity and coupling control efficiency during several substrate states induced via separate titrations. All titrations were added in series as presented, whereby the concentrations of substrates, uncouplers and inhibitors used were based on prior experiments [24]. The titration protocol was modified from previous protocols where they are described in detail [24]. In short, leak respiration in absence of adenylates (L_N_) was induced with the addition of octanoyl carnitine (0.2 mM) and malate (2 mM). Specifically, L_N_ represents the resting O_2_ consumption of an unaltered and intact electron transport system (ETS) free of adenylates. Maximal electron flow through electron-transferring flavoprotein and maximal fatty acid oxidative capacity (P_ETF_) was subsequently determined following the addition of ADP (5 mM). Electron capacity through complex I (P_CI_) was then induced following the additions of pyruvate (5 mM) and glutamate (10 mM). Maximal oxidative phosphorylation capacity (P) was induced with the addition of succinate (10 mM). P thereby represents respiration that results from saturating concentrations of ADP and substrate supply both for complex I and II. As an internal control for the integrity of the mitochondrial preparation, the mitochondrial outer membrane was assessed with the addition of cytochrome C (10 μM). ATP synthase was then inhibited by the titration of oligomycin (1 μM), which leads to oligomycin-induced leak respiration (L_Omy_). L_Omy_ represents the corresponding leak state to P. In L_Omy_ the chemiosmotic gradient is at maximum because of maximal substrate supply and inhibition of complex V (ATP synthase). Additionally, O_2_ flux is at minimum and is representative of proton leak, slip, cation cycling and overall dyscoupling. By uncoupling ATP synthase from the electron transport chain with the step-wise titration (4 × 0.5 μM) of the proton ionophore carbonyl cyanide *p*-(trifluoromethoxy) phenylhydrazone (FCCP) phosphorylative restraint of electron transport was assessed reaching ETS capacity (E). In order to inhibit CI and to assess electron flow specific to complex II (P_CII_), rotenone (0.5 μM) was added. P_CII_ is thereby not influenced by the preceding addition of FCCP, which was verified with separate protocols in our laboratory. The addition of antimycin A (2.5 μM) that inhibits complex III allows the determination and correction of residual O_2_ consumption (ROX), which is indicative of non-mitochondrial O_2_ consumption in the chamber. Finally, respiration measurements were terminated by simultaneous titration of ascorbate and TMPD to assess complex IV (COX) activity. Ascorbate and TMPD represent redox substrates that donate electrons directly to COX. Correction of O_2_ flux for the side reaction of auto-oxidation was conducted by chemical calibration experiments prior to the measurements. Finally, mitochondrial leak control ratios (LCR) were analysed as well. LCR allow the description of mitochondrial coupling efficiency, with a theoretical minimum of 0.0, indicating a fully coupled system, to a value of 1.0, representing a fully uncoupled system [24, 25].

### Transmission electron microscopy

The chemically fixed tissue was washed 3 × in 0.1. M cacodylate buffer and consecutively post-fixed in 1 % osmiumtetroxid in 0.1. M cacodylate buffer for 2 h at RT. After 3 x washing with milliQ H_2_O, samples were block-stained with uranyl acetate (2 % in milliQ H_2_O) overnight at RT. The subsequent graded dehydration was conducted in a tissue processor (Leica EM TP, Leica Microsystems, Wetzlar, Germany) according to the following protocol: 10 min 70 % EtOH, 15 min 96 % EtOH, 4 × 30 min 100 % EtOH, 2 × 5 min and 2 × 10 min propylene oxide, 2 × 1 h 1:1 propylene oxide/Epon, 1 h 100 % Epon, 100 % Epon over night, 3 h Epon. Finally, samples were embedded in 100 % Epon in a non-oriented (isotropic) way and Epon was polymerised for 48 h at 60 °C. Ultrathin sections were cut on an ultramicrotome (Ultracut E ultramicrotome, Reichert, NY, USA) with a diamond knife (Diatome, Biel, Switzerland) and placed on 50 mesh hexagonal cupper grids (Plano GmbH, Wetzlar, Germany). Specifically, from each block three ultrathin sections (70 nm) were obtained at two depths separated by 20 μm and placed onto two grids. Finally, the grids were contrasted with lead citrate [26]. Micrographs were obtained in a FEI Tecnai G2 Spirit electron microscope (Tecnai G2 Spirit, FEI, Hillsboro, OR, USA) mounted with an Orius SC1000 charged-coupled device camera (Gatan, Pleasanton, GA, USA) and interfaced with the TEM software (TEM User Interface, FEI, Hillsboro, OR, USA). Micrographs were acquired in a uniform random systematic order. For each grid three predetermined positions were marked for further imaging. At each position an area of 222 × 146 μm^2^ (pixel size of 57.7 nm) was used for automated image capturing by the TEM photomontage software. A random starting point was selected for the first micrograph whereafter further micrographs were taken at fixed x-, y-intervals of 64 and 42 μm, respectively. Nine 15.9 × 10.5 μm^2^ micrographs (3840 × 2528 pixels) were captured in each area. Consequently, for each biopsy a total of 54 micrographs were acquired (nine micrographs per three positions per two sections per sample). Subsequently, skeletal muscle volume density of mitochondria (Mito_VD_) was estimated by point counting in combination with Cavalieri’s principle [27] using the Stereo-Investigator software (MBF Bioscience, Williston, ND, USA). For point counting a grid spacing of 1 μm along bot x- and y-axis was applied. Each point was assigned as either intermyofibrilar (IMF_VD_) or subsarcolemmal (SS_VD_) mitochondrial volume density, lipid droplet volume density (LD_VD_), skeletal muscle or “nothing”. SS mitochondria were defined as the mitochondria that were not separated by myofibrils from the sarcolemma.

### Histochemistry

Consecutive transverse sections (8 μm) were cut at three depths on a microtome (Leica CM 1850, Leica Biosystems, Wetzlar, Germany) at −22 °C, mounted on glass cover slides (Superfrost Plus, Thermo Fisher Scientific Inc., Rockford, IL, USA), set to air dry and stored at -20 °C until further processing. The serial sections were then fixed in 3 % neutral buffered formalin at RT for 45 min, briefly washed and blocked with 5 % goat serum. Thereafter, the sections were firstly incubated with a primary antibody against myosin heavy chain (MyHC) isoform I (Novocastra Lyophilized Mouse Monoclonal Antibody Myosin Heavy Chain (slow), NCL-MHCs, Leica Biosystems, Wetzlar, Germany) and secondly with goat anti-rabbit IgG secondary antibody conjugated with Alexa Fluor 488 (Thermo Fisher Scientific Inc., Rockford, IL, USA). The sarcolemma of the skeletal muscle fibers were visualised by incubation with a primary antibody against laminin (Novocastra Lyophilized Mouse Monoclonal Antibody Laminin, NCL-LAMININ, Leica Biosystems, Wetzlar, Germany) in combination with goat anti-rabbit IgG secondary antibody, Alexa Fluor 647 conjugate (Thermo Fisher Scientific Inc., Rockford, IL, USA). An automated upright microscope system was used for digitizing the sections (Leica, DM5500 B, Leica Microsystems, Wetzlar, Germany). Skeletal muscle fiber type distribution was determined according to their MyHC-I and MyHC-II isoforms and classified into type I and type II fibers. For all analyses, only fibers fully encircled by adjacent fibers were evaluated using Adobe Photoshop Pro CS6 (Adobe Systems Incorporated, San Jose, CA, USA). Fiber cross-sectional area (CSA) was determined by encircling the boundaries of the muscle cells of at least 50 fibers per fiber type. Only fibers with a circularity higher than 0.7 were considered for analysis (perfect circle = 1.0).

### Statistical analysis

All data are presented as mean ± SD in text and figures. The statistical analysis was conducted using the software SPSS Statistics 22.0 (SPSS, Chicago, IL, USA). After verification of normal distribution of the data, two-tailed unpaired samples *t*-tests were conducted to test the null hypothesis stating no difference between patients with mitochondrial myopathy and healthy controls. For all statistical analyses, a value of *P* < 0.05 was considered significant. To evaluate the effect sizes of the important findings Cohen’s d were calculated, whereby d < 0.3 is considerd a small effect, d > 0.3 and < 0.8 a medium effect and d > 0.8 a large effect. Spearman’s rank correlation coeffiencts were calculated to determine potential dependences of several variables. The number of patients and controls included in each data set are always indicated and can vary due to inability of cycling above a power output of 0 W (one case) or technical problems with the devices (two cases).

## Results

All participants completed the skeletal muscle biopsy procedure and DXA measurement. Except from one patient who was not able to cycle against a certain resistance (25 W), all participants conducted the incremental and the constant-load cycling exercise test. Due to technical reasons, one patient and one control could not perform the isokinetic dynamometry test. There were no group differences for age, body composition, resting blood lactate concentration, t_lim_ and MVC (Table 1).

### Exercise performance

*P*_peak_ was 53 % lower (*P* < 0.05, d = 1.8) in patients with mitochondrial myopathy. In accordance, *V̇*O_2peak_ was 45 % lower (*P* < 0.05, d = 1.8) in mitochondrial myopathy patients than in healthy controls, with a broad range in both groups (Table 1). However, RER at the same absolute and relative power output did not differ between groups, suggesting similar metabolic regulations in patients and controls at submaximal work rates. Additionally, t_lim_ in the constant-load test did not differ between groups.

### Skeletal muscle mitochondrial volume density and respiratory capacity

**Fig. 1:**
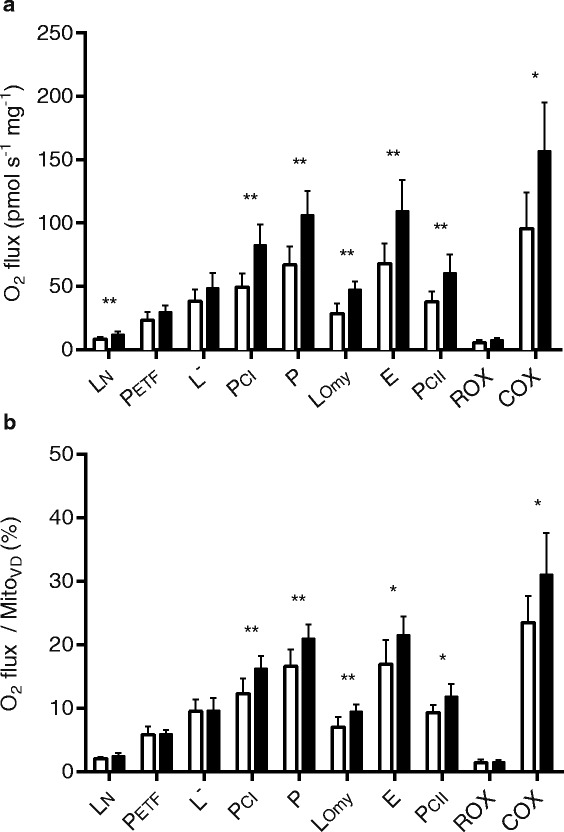
Mass-specific mitochondrial (**a**) and mitochondrial-specific (**b**) respiratory capacity (normalised to Mito_VD_). Respiratory capacities are presented for patients with mitochondrial myopathy in white bars and healthy controls in black bars) L_N_, leak respiration without adenylates; P_ETF_, fatty acid oxidative capacity; P_CI_, respiratory capacity of complex I; P, oxidative phosphorylation capacity; L_Omy_, oligomycin-induced leak respiration; E, electron transport system capacity; P_CII_, respiratory capacity of complex II; ROX, residual oxygen consumption; COX, respiratory capacity of complex IV. Values are mean ± SD. *P < 0.05; **P < 0.01. n_Patients_ = 6, n_Controls_ = 8

Maximal mitochondrial respiration specific to each individual mitochondrial complex (mass-specific respiration, respiration per mg ww) was lower (*P* < 0.05) in patients with mitochondrial myopathy vs. healthy controls (Fig. 1a). In particular, difference in mass-specific respiratory capacity with mitochondrial myopathy amounted to 50 (P_CI_, d = 2.3), 46 (P_CII_, d = 1.8), 45 (P, d = 2.3), 48 (COX, d = 1.8) and 47 % (ETS, d = 2.0). Considering the range of P for example, patients show a markedly lower maximal mitochondrial oxidative capacity (44.10 – 81.94 pmol O_2_ mg^−1^ s^−1^) than controls (86.54 – 127.89 pmol O_2_ mg^−1^ s^−1^; *P* < 0.01). Additionally, Mito_VD_ was on average 23 % lower (*P* < 0.05, d = 1.5) in patients, which was related to the lower IMF_VD_ (Table 3, d = 1.4). Moreover, after normalizing mitochondrial respiratory capacity to Mito_VD_ all respiratory states specific to the five mitochondrial complexes were lower (*P* < 0.05, Fig. 1b) in patients (28 (P_CI_, d = 1.9), 24 (P_CII_, d = 1.5), 23 (P, d = 1.8), 28 (COX, d = 1.3) and 24 % (ETS, d = 1.4)), suggesting quantitative and qualitative alterations in mitochondrial function with mitochondrial myopathy. In contrast, LCR did not differ (*P* = 0.501) between groups (mitochondrial myopathy 0.42 ± 0.07; controls 0.44 ± 0.05).

**Table 3:** Skeletal muscle properties of patients with mitochondrial myopathy (*n* = 6) and healthy controls (*n* = 8)

	Patients	Controls	*P*-value
Type I fibers (%)	34.8 ± 3.6	55.7 ± 5.9	< 0.001
Type II fibers (%)	65.2 ± 3.6	44.3 ± 5.9	< 0.001
CSA total (μm^2^)	3,438 ± 915	4,698 ± 1,328	0.070
CSA type I fibers (μm^2^)	3991 ± 1211	5234 ± 1566	0.133
CSA type II fibers (μm^2^)	3,178 ± 852	4,359 ± 1,592	0.127
Mito_VD_ (%)	4.0 ± 0.5	5.1 ± 0.8	< 0.05
IMF_VD_ (%)	3.4 ± 0.4	4.1 ± 0.5	< 0.05
SS_VD_ (%)	0.6 ± 0.3	1.0 ± 0.5	0.118
LD_VD_ (%)	0.4 ± 0.1	0.4 ± 0.3	0.660

Values are represented as means ± SD. *CSA* cross sectional area; *IMF*_*VD*_ intermyofibrilar mitochondrial volume density, *LD*_*VD*_ lipid droplet volume density, *Mito*_*VD*_ mitochondrial volume density, *SS*_*VD*_ subsarcolemmal mitochondrial volume density

### Skeletal muscle fiber type distribution

Mitochondrial myopathy patients demonstrated a higher (*P* < 0.001, d = 4.2) ratio of type II to I fibers and a tendency (*P* = 0.070, d = 1.1) towards lower CSA, indicating potential skeletal muscle atrophy (Table 3). In accordance with these results, the distribution of type II fibers in patients ranged from 60.4 – 69.0 % in contrast to 32.8 – 48.8 % in healthy controls. However, CSA specific to type I and II fibers did not differ (*P* = 0.133; *P* = 0.127) between patients and controls (Table 3).

### Correlations

The mutation load in the 6 patients ranged from 12 to 95 %. There was no relationship between percentage mutation and any of the other determined variables. *V̇*O_2peak_ was positively correlated (*P* < 0.05) to P (*ρ* = 0.626), Mito_VD_ (*ρ* = 0.577) and P normalised to Mito_VD_ (*ρ* = 0.648)_._ Moreover, P, Mito_VD_, P/Mito_VD_ as well as lean mass were all related to CSA of type I fibers (*ρ* = 0.653, *ρ* = 0.600, *ρ* = 0.618, *ρ* = 0.578; *P* < 0.05).

## Discussion

The main finding of the present study is that skeletal muscle mitochondrial function in mitochondrial myopathy patients is lower than in healthy controls due to reduced mitochondrial volume density as well as diminished intrinsic mitochondrial function. Furthermore, patients exhibited a shift in skeletal muscle fiber type towards more type II fibers and tended to an atrophic muscle phenotype. Moreover, *V̇*O_2peak_ and *P*_peak_ in patients with mitochondrial myopathy were lower than in healthy controls. Mitochondrial volume density and maximal oxidative phosphorylation capacity were positively correlated to *V̇*O_2peak._ Thus, mitochondrial myopathy is associated with decreased mitochondrial quantity and quality as well as diminished capacity for whole-body maximal O_2_ uptake.

This study determined skeletal muscle mitochondrial volume density and respiratory capacity of each single complex I-V in permeabilised fibers from mitochondrial myopathy patients in comparison to age- and gender-matched healthy controls. Thereby, not only Mito_VD_ but also the mass-specific (per mg ww) and mitochondrial-specific (normalised to Mito_VD_) respiratory capacity of each individual complex were affected by mitochondrial myopathy, indicating an evident impairment of innate mitochondrial function. This lower mitochondrial function may lead to greater disturbances in homeostasis in skeletal muscle, higher reliance on muscle glycogen, increased lactate production and, therewith, lower exercise capacity compared to healthy individuals [28]. Moreover, these alterations could limit the skeletal muscle’s ability to extract and/or oxidize O_2_ from the blood [14, 28], potentially resulting in an impaired *V̇*O_2peak_. Particularly, the lower O_2_ extraction capacity of the skeletal muscle is likely to lead to a higher systemic O_2_ delivery relative to O_2_ utilization, i.e. a hyperkinetic circulation, causing typical symptoms of mitochondrial myopathy such as metabolic acidosis, exertional dyspnea, exercise intolerance, low exercise performance and consecutively reduced quality of life [10–13, 29].

Hence, the present bioenergetic impairments at the cell organelles’ level result in abnormal cardiac and ventilatory responses to increased O_2_ requirements as in response to physical activity and exercise [29–31], and might impair exercise capacity in patients with mitochondrial myopathy. Noteworthy, exercise capacity in patients with mitochondrial myopathy was lower compared to healthy controls and varied broadly, reflecting the heterogenous clinical manifestation and severity of this disease. In addition, *V̇*O_2peak_ correlated with measures of mitochondrial quantity and quality, indicating an association with reduced skeletal muscle O_2_ uptake capacity, which has been indicated by previous studies in our laboratory before [32, 33]. In support, diminished *V̇*O_2peak_ may be entirely explained by a lower systemic a-vO_2_ diff [10–13], since peak cardiac output (Q̇_peak_) has been reported not to differ between mitochondrial myopathy and healthy controls [11]. Although these metabolic adaptations in skeletal muscle are critical for physical activity, especially for submaximal exercise performance, *V̇*O_2peak_ is limited by O_2_ transport capacity in healthy human individuals whithout metabolic disturbances [34–36].

To our knowledge, this is also the first study demonstrating skeletal muscle fiber type transformation from type I to type II fibers in adult mitochondrial myopathy patients. This finding contrasts to the histochemically described type I fiber predominance as a compensation for impaired energy production and mitochondrial function in children with mitochondrial myopathy [8]. As type I fibers usually contain a higher proportion of mitochondria than type II fibers [37], an enhanced ratio of type I as compared to type II fibers could increase the number of mitochondria and lead to a potentially greater capacity for energy production [8]. In turn, decreased oxidative capacity could also be compensated by a switch from an oxidative to a more glycolytic phenotype in order to partially restore muscle strength and energy production, yet leading to higher lactic acid production, which is characteristic for this disease [10, 38]. Lactate in turn represents an important fuel for oxidative metabolism and has been reported to rather enhance energy production than inducing fatigue in patients with mitochondrial myopathy [39]. In support of our findings, Venhoff et al. [9] reported a fiber type shift from type I to type II fibers in rodent models of mitochondrial myopathy. The lower Mito_VD_ in patients with mitochondrial myopathy, which was related to lower IMF_VD_, may thereby reflect the decreased type I to type II fiber ratio and the potentially higher reliance on glycogen metabolism. SS_VD_ in the present study did not differ and only half of the mitochondrial myopathy patients demonstrated ragged-red fibers (RRF, Table 2). Together these findings contrast previous reports of SS mitochondrial proliferations and simulatenously occurring RRF with mitochondrial myopathy [8, 40, 41]. Moreover, mouse models of mitochondrial myopathy demonstrated RRF and increased skeletal muscle mitochondrial volume density, particularly in the SS region, wheras respiratory chain enzyme activites were decreased [42]. Therefore, the authors suggested the higher mitochondrial volume density to partly compensate for the respiratory chain deficiency and the redcued mitochondrial ATP production being not as critical for the pathophysiology of mitochondrial myopathy as previously thought [42]. Accordingly, recent studies displayed altered Ca^2+^ handling in mitochondrial myopathy [43, 44], stimulating the discussion of progressive muscle weakness rather than energy deficiency as the potential dominating pathomechanism [44]. Collectively, these partially divergent findings may once more reflect the heterogenous character of this disease and the possible detrimental effect of physical inactivity in mitochondrial myopathy patients, which in turn emphasizes the need for further studies also on alternate pathophysiological mechanisms.

Discriminating exercise intolerance caused by defective mitochondria from that of disease-associated inactivity and hence physical deconditioning remains challenging. Exercise intolerance inevitably results in reduced levels of habitual physical activity and causes physical deconditioning, which in turn leads to a vicious cycle of further deconditioning and progressive exercise intolerance [45]. Similar total lean mass and lean mass of the lower limbs in mitochondrial myopathy patients and healthy controls may suggest that the diminished mitochondrial volume density, as well as the reduced function and exercise performance are possibly associated with the disease itself rather than with inactivity alone. Moreover, MVC and t_lim_ did not significantly differ between patients with mitochondrial myopathy and healthy controls, which contradicts different levels of physical activity. In support, mice with mitochondrial myopathy showed a decrease in ATP levels even with exercise, which was less than in sedentary diseased mice but dropped to 30 % of wild-type after 10 month [46]. Nonetheless, in spite of matching patients and controls according to age, gender and, as far as possible, to reported physical activity levels, the lower *V̇*O_2peak_ and *P*_peak_ in patients with mitochondrial myopathy could be due to sustained physical inactivity associated with the disease-related exercise intolerance. Thereby, the present lower Mito_VD_ and mitochondrial respiratory capacity in patients with mitochondrial myopathy could indeed also simply be a reflection of this vicious cycle. Disuse and physical inactivity, respectively, have been reported to lead to decreases in mitochondrial volume density and hence mitochondrial function [47, 48]. In line herewith is the lower IMF_VD_ with mitochondrial myopathy, as IMF mitochondria represent a specialization towards energy production for contractile activity [49]. Reduced contractile activity due to physical inactivity in patients with mitochondrial myopathy could have resulted in lower IMF_VD_ and secondary impaired mitochondrial function. In support, muscle homogenates of mice with mitochondrial myopathy due to a COX deficiency exhibited increased mitochondrial mass, oxidative capacity and exercise performance after an endurance exercise training intervention in comparison to sedentary diseased mice [46]. However, although the enhanced physical activity led to a preservation of oxidative capacity of 50 – 60 % of wild-type levels in comparison to the drop to 10 – 40 % in sedentary diseased mice in 3 month duration, oxidative capacity still remained reduced compared to wild-type controls. Moreover, mitochondrial COX activity was not affected by the exercise intervention [46]. The oxidative capacity in muscle homogenates, which was sustained only partially, together with the persistent COX defect at the mitochondrial level in exercised diseased mice indicates increased mitochondrial quantity but not quality with exercise [46]. Therefore, lower Mito_VD_ but not impaired intrinsic mitochondrial function could be explained by physical inactivity secondarily to exercise intolerance. Congruently, two weeks of physical inactivity induced by one leg immobilization led to reduced mitochondrial respiratory capacity due to changes in mitochondrial content but not intrinsic mitochondrial function [47]. Moreover, considering the shift towards more type II fibers and the trend to general fiber atrophy, physical inactivity should be considered as a potential cause. However, muscle disuse affects mainly type I fiber diameter [50, 51], which did not differ between patients and controls. This is further supported by a rat model of mitochondrial myopathy harborating a predominate type II fiber atrophy [9]. Conclusively, mechanisms related to mitochondrial dysfunction and the disease-causing mutations itself could have induced the present pathologic findings. However, physical inactivity could secondarily have led to further aggraviation of decreased exercise capacity and mitochondrial function. Therefore, the present study cannot definitively clarify whether the findings are related to the disease per se or in addition to inacitivity. Another limitation of the present study is the small sample size, which hovewer, according to the preceding power calculation and the significant differences of the main results was sufficient. The small number of participants can be explained by the large expected contrasts in Mito_VD_ and mitochondrial respiratory capacity between patients with mitochondrial myopathy and healthy controls and the small coefficients of variation for the TEM and high-resolution respirometry. The present findings in this small cohort with a rather inhomogenous degree of heteroplasmy are further strengthend by the high effect sizes.

From a clinical point of view, the diagnosis of mitochondrial cytopathy or mitochondrial myopathy, respectively, remains challenging and requires the combined application of multiple methodologies [52]. Blood samples may exhibit increased resting lactate concentrations and elevated CK levels. However, lactic acidosis is often not present and CK levels may be normal or only mildly increased. Functional muscle testing with the subanaerobic threshold exercise test (SATET) is a very specific measure for mitochondrial pathology [53] but many mitochondrial myopathy patients do not exhibit pathological values. Morphological, biochemical and molecular studies in tissue samples allow a diagnosis by the presence of suggestive histopathological findings such as RRF and ragged-blue fibers, COX depleted fibers, variously formed mitochondria, abnormal cristae, crystalloid inclusions and the determination of disease-causing mutations in the mitochondrial and/or nuclear DNA [41, 52]. As demonstrated in two of our six patients, these typical histological signs of mitochondrial myopathy can be lacking despite presence of a mitochondrial disease [52]. Molecular genetic analysis of mitochondrial DNA in tissues may demonstrate heteroplasmic deletions and point mutations. In addition, nuclear DNA mutations need to be considered. For heteroplasmic mitochondrial DNA mutations, the degree of heteroplasmy needs to be quantified, but the pathological threshold is poorly defined, particularly in mitochondrial cytopathy patients at advanaced age. This heteroplasmy, a canonical criteria for the pathogenity, is not always given as also pathogenic homoplasmic mutations have been described [3, 54]. This aspect also causes some limitations regarding the results of the present study and their generalization for all mitochondrial myopathy patients. As we included only patients with mitochondrial DNA single deletions, the present results are only true for this spectrum of phenotype and further investigations considering patients with mitochondrial DNA point mutations and nuclear DNA mutations affecting mitochondria are needed to verify these findings.

To provide proper patient management, treatment and genetic counseling, a clear diagnosis of mitochondrial cytopathies is necessary. Our results demonstrate that high-resolution respirometry measurements could represent a promising additional tool for the diagnosis of mitochondrial cytopathies. In vitro measurements as the present high-resolution respirometry allow the estimation of the functionality of specific steps implicated in mitochondrial metabolism by the use of various substrates, uncouplers and inhibitors. Such polarographic measurements of O_2_ consumption provide more essential information on innate mitochondrial function, as evaluations not only of isolated mitochondria but also of permeabilised cells are possible [55]. In contrast to the isolation procedure, permeabilization of muscle fibers preserves mitochondrial morphology and integrity and allows the examination of the intact mitochondrial network [22]. However, the definition of normal ranges of complex activites in skeletal muscle remains disputed and difficult. Since a skeletal muscle biopsy is needed for this approach, this minimal-invasive procedure represents a disadvantage for the patients. Conclusively, further studies to eliminate the variations and to validate high-resolution respirometry as a diagnostic measure are needed.

## Conclusion

Mitochondrial myopathy led to diminished skeletal muscle mitochondrial volume density and respiratory capacity of each individual complex and was related to reduced maximal exercise capacity. Lower mitochondrial volume density was attended by a shift in skeletal muscle phenotype from type I to type II fibers, reflecting a possible compensation for the lower mitochondrial respiratory as well as exercise capacity. Hence, the affected mitochondrial quantity and quality could be responsible for the impaired exercise capacity and could further aggravate exercise intolerance. However, it cannot be excluded that changes observed in patients are simply due to physical inactivity as a cause of the disease-related exercise intolerance. Nonetheless, the clearly reduced mitochondrial respiratory capacity in patients with mitochondrial myopathy compared to age- and gender-matched healthy controls proposes high-resolution respirometry measurements in permeabilised skeletal muscle fibers to be a promising additional diagnostic tool.

## Abbreviations

BMC, bone mineral content; CIS-fatigue, checklist individual strength; CK, phosphocreatine kinase; COX, complex IV activity; CSA, cross-sectional area; DXA, dual-energy X-ray absorptiometry; E, electron transport system capacity; ETS, electron transport system; FCCP, carbonyl cyanide *p*-(trifluoromethoxy) phenylhydrazone; HbA_1c_, glycated hemoglobin; IMF_VD_, intermyofibrilar mitochondrial volume density; LCR, leak control ratio; LD_VD_, lipid droplet volume density; L_N_, leak respiration in absence of adenylates; L_Omy_, oligomycin-induced leak respiration; Mito_VD_, volume density of mitochondria; MVC, maximal voluntary contraction torque; P, maximal oxidative phosphorylation capacity; P_CI_, complex I activity; P_CII_, complex II activity; P_ETF_, fatty acid oxidative capacity; *P*_peak_, work capacity; proBNP, precursorprotein brain natriuretic peptide; Q̇_peak_, peak cardiac output; RER, respiratory exchange ratio; ROX, residual O_2_ consumption; rpm, revolutions per minute; RT, room temperature; SS_VD_, subsarcolemmal mitochondrial volume density; TEM, transmission electron microscopy; t_lim_, time to volitional exhaustion; TSH, thyroid stimulating hormone; VAS, visual analogue scale; *V̇*O_2peak_, peak oxygen uptake; ww, wet weight
